# Supplementary material for: Quantum annealing for enhanced feature selection in single-cell RNA sequencing data analysis
Source: Quantum Mach Intell. Author manuscript; Available in PMC 2026 Mar 25. (PMC13012334; doi:10.1007/s42484-025-00312-1)
Supplement: ESM1 [file NIHMS2140852-supplement-ESM1.docx]

# Supplementary Information: Quantum Annealing for Enhanced Feature Selection in Single-Cell RNA Sequencing Data Analysis-Supplemental Information

Selim Romero^1,2,3^, Shreyan Gupta^1,3^, Victoria Gatlin^1,3^, Robert S. Chapkin^2,3^, and James J. Cai^1,3,4*^

^1^Department of Veterinary Integrative Biosciences, Texas A&M University, College Station, TX 77843, USA.

^2^Department of Nutrition, Texas A&M University, College Station, TX 77843, USA.

^3^CPRIT Single Cell Data Science Core, Texas A&M University, College Station, TX 77843, USA.

^4^Department of Electrical and Computer Engineering, Texas A&M University, College Station, TX 77843, USA.

This document contains the supplementary materials for the accompanying research paper, including Enrichr analysis, the features selected by each method, and figures specifically related to the QUBO feature selection process.

**Supplementary Table 1. Enrichr Pathways of QUBO-Selected Genes in hESC-EC Differentiation.** Significant Enrichr pathway analysis results for genes selected by the QUBO method in the hESC-EC differentiation dataset are shown below.

| **GO Biological Process 20223** | | | | |
| --- | --- | --- | --- | --- |
| **Term** | **Overlap** | **P-value** | **Adj.**  **P-value** | **Genes** |
| Negative Regulation Of Signal Transduction (GO:0009968) | 5/267 | 5.30E-04 | 1.34E-01 | SFRP1;IGFBP5;YWHAB;GDF15;APLN |
| Positive Regulation Of Focal Adhesion Assembly (GO:0051894) | 2/18 | 9.13E-04 | 1.34E-01 | SFRP1;THY1 |
| Response To Fibroblast Growth Factor (GO:0071774) | 2/20 | 1.13E-03 | 1.34E-01 | SFRP1;IER2 |
| Positive Regulation Of Cell-Substrate Junction Organization (GO:0150117) | 2/23 | 1.50E-03 | 1.34E-01 | SFRP1;THY1 |
| Cellular Response To Transforming Growth Factor Beta Stimulus (GO:0071560) | 3/96 | 1.78E-03 | 1.34E-01 | SFRP1;GDF15;ID1 |
| Negative Regulation Of Neurogenesis (GO:0050768) | 2/27 | 2.07E-03 | 1.34E-01 | DYNLT1;THY1 |
| Negative Regulation Of Peptidyl-Tyrosine Phosphorylation (GO:0050732) | 2/30 | 2.55E-03 | 1.34E-01 | SFRP1;THY1 |
| Regulation Of T Cell Receptor Signaling Pathway (GO:0050856) | 2/34 | 3.27E-03 | 1.34E-01 | CD81;THY1 |
| Positive Regulation Of DNA-templated Transcription (GO:0045893) | 9/1243 | 3.34E-03 | 1.34E-01 | ACTR2;SFRP1;BEX1;CD81;ATF5;LITAF;IER2;HMGN1;APLN |
| Negative Regulation Of Smooth Muscle Cell Proliferation (GO:0048662) | 2/37 | 3.86E-03 | 1.34E-01 | IGFBP5;APLN |
| Regulation Of Vascular Associated Smooth Muscle Cell Proliferation (GO:1904705) | 2/37 | 3.86E-03 | 1.34E-01 | IGFBP5;APLN |
| Negative Regulation Of Cell Motility (GO:2000146) | 3/133 | 4.49E-03 | 1.34E-01 | SFRP1;IGFBP5;THY1 |
| Positive Regulation Of Catalytic Activity (GO:0043085) | 2/40 | 4.50E-03 | 1.34E-01 | YWHAB;XRCC5 |
| DNA Recombination (GO:0006310) | 2/42 | 4.95E-03 | 1.34E-01 | XRCC5;HMGB3 |
| Positive Regulation Of Cell-Matrix Adhesion (GO:0001954) | 2/43 | 5.18E-03 | 1.34E-01 | SFRP1;THY1 |
| Negative Regulation Of Nucleic Acid-Templated Transcription (GO:1903507) | 5/456 | 5.50E-03 | 1.34E-01 | SFRP1;XRCC5;ID1;ID3;ATF5 |
| Bicellular Tight Junction Assembly (GO:0070830) | 2/47 | 6.16E-03 | 1.34E-01 | RAMP2;CLDN7 |
| Receptor Internalization (GO:0031623) | 2/51 | 7.22E-03 | 1.34E-01 | RAMP2;CD81 |
| Tight Junction Assembly (GO:0120192) | 2/53 | 7.78E-03 | 1.34E-01 | RAMP2;CLDN7 |
| Blood Vessel Morphogenesis (GO:0048514) | 2/53 | 7.78E-03 | 1.34E-01 | RAMP2;ID1 |
| Negative Regulation Of Cell Migration (GO:0030336) | 3/163 | 7.87E-03 | 1.34E-01 | SFRP1;IGFBP5;THY1 |
| Regulation Of Focal Adhesion Assembly (GO:0051893) | 2/54 | 8.07E-03 | 1.34E-01 | SFRP1;THY1 |
| Apical Junction Assembly (GO:0043297) | 2/54 | 8.07E-03 | 1.34E-01 | RAMP2;CLDN7 |
| Regulation Of Smooth Muscle Cell Proliferation (GO:0048660) | 2/54 | 8.07E-03 | 1.34E-01 | IGFBP5;XRCC5 |
| Response To Organic Cyclic Compound (GO:0014070) | 2/58 | 9.26E-03 | 1.34E-01 | SFRP1;IGFBP5 |
| Positive Regulation Of Hydrolase Activity (GO:0051345) | 3/176 | 9.70E-03 | 1.34E-01 | SFRP1;RGS10;THY1 |
| Negative Regulation Of Gene Expression (GO:0010629) | 4/336 | 9.82E-03 | 1.34E-01 | SFRP1;IGFBP5;NTS;APLN |
| Positive Regulation Of Cell Junction Assembly (GO:1901890) | 2/61 | 1.02E-02 | 1.34E-01 | SFRP1;THY1 |
| Negative Regulation Of Macromolecule Metabolic Process (GO:0010605) | 3/186 | 1.13E-02 | 1.34E-01 | SFRP1;NTS;APLN |
| Regulation Of Cell-Matrix Adhesion (GO:0001952) | 2/65 | 1.15E-02 | 1.34E-01 | SFRP1;THY1 |
| Regulation Of Cell Population Proliferation (GO:0042127) | 6/766 | 1.17E-02 | 1.34E-01 | SFRP1;CD81;XRCC5;NAP1L1;  ATF5;TP53I11 |
| Regulation Of Neurogenesis (GO:0050767) | 2/67 | 1.22E-02 | 1.34E-01 | DYNLT1;NAP1L1 |
| Protein Localization To Membrane (GO:0072657) | 3/192 | 1.23E-02 | 1.34E-01 | RAMP2;CD81;THY1 |
| **KEGG 2021 Human** | | | | |
| **Term** | **Overlap** | **P-value** | **Adj.**  **P-value** | **Genes** |
| Tight junction | 5/169 | 6.33E-05 | 4.93E-03 | TUBA1C;ACTR2;ARPC3;CLDN7;MYL12B |
| **Reactome 2022** | | | | |
| **Term** | **Overlap** | **P-value** | **Adj.**  **P-value** | **Genes** |
| Signal Transduction R-HSA-162582 | 16/2465 | 2.15E-04 | 5.00E-02 | ACTR2;RAMP2;YWHAB;TRH;NTS;MYL12B;APLN;  DRAP1;SFRP1;GNG5;ARPC3;ID1;EIF4EBP1;RGS10;ID3;VIM |
| Response Of EIF2AK1 (HRI) To Heme Deficiency R-HSA-9648895 | 2/15 | 6.30E-04 | 7.34E-02 | EIF2S2;ATF5 |
| EPH-Ephrin Signaling R-HSA-2682334 | 3/91 | 1.53E-03 | 8.46E-02 | ACTR2;ARPC3;MYL12B |
| mTORC1-mediated Signaling R-HSA-166208 | 2/24 | 1.63E-03 | 8.46E-02 | YWHAB;EIF4EBP1 |
| Signaling By NTRK1 (TRKA) R-HSA-187037 | 3/114 | 2.91E-03 | 8.46E-02 | YWHAB;ID1;ID3 |
| EPHB-mediated Forward Signaling R-HSA-3928662 | 2/34 | 3.27E-03 | 8.46E-02 | ACTR2;ARPC3 |
| Leishmania Infection R-HSA-9658195 | 4/247 | 3.35E-03 | 8.46E-02 | ACTR2;RAMP2;GNG5;ARPC3 |
| RHO GTPases Activate WASPs And WAVEs R-HSA-5663213 | 2/36 | 3.65E-03 | 8.46E-02 | ACTR2;ARPC3 |
| NGF-stimulated Transcription R-HSA-9031628 | 2/39 | 4.28E-03 | 8.46E-02 | ID1;ID3 |
| GPCR Downstream Signaling R-HSA-388396 | 6/619 | 4.29E-03 | 8.46E-02 | RAMP2;GNG5;RGS10;TRH;NTS;APLN |
| Signaling By NTRKs R-HSA-166520 | 3/132 | 4.39E-03 | 8.46E-02 | YWHAB;ID1;ID3 |
| RHO GTPase Effectors R-HSA-195258 | 4/269 | 4.54E-03 | 8.46E-02 | ACTR2;YWHAB;ARPC3;MYL12B |
| MTOR Signaling R-HSA-165159 | 2/41 | 4.72E-03 | 8.46E-02 | YWHAB;EIF4EBP1 |
| GPCR Ligand Binding R-HSA-500792 | 5/458 | 5.60E-03 | 9.31E-02 | RAMP2;GNG5;TRH;NTS;APLN |
| Signaling By GPCR R-HSA-372790 | 6/689 | 7.16E-03 | 1.11E-01 | RAMP2;GNG5;RGS10;TRH;NTS;APLN |
| mRNA Activation Upon Binding Of Cap-Binding Complex And eIFs, Subsequent Binding To 43S R-HSA-72662 | 2/58 | 9.26E-03 | 1.23E-01 | EIF4EBP1;EIF2S2 |
| FCGR3A-mediated Phagocytosis R-HSA-9664422 | 2/60 | 9.88E-03 | 1.23E-01 | ACTR2;ARPC3 |
| Nuclear Events (Kinase And Transcription Factor Activation) R-HSA-198725 | 2/61 | 1.02E-02 | 1.23E-01 | ID1;ID3 |
| RHO GTPases Activate PKNs R-HSA-5625740 | 2/62 | 1.05E-02 | 1.23E-01 | YWHAB;MYL12B |
| Regulation Of Actin Dynamics For Phagocytic Cup Formation R-HSA-2029482 | 2/62 | 1.05E-02 | 1.23E-01 | ACTR2;ARPC3 |
| Semaphorin Interactions R-HSA-373755 | 2/64 | 1.12E-02 | 1.24E-01 | DPYSL2;MYL12B |
| Peptide Ligand-Binding Receptors R-HSA-375276 | 3/196 | 1.30E-02 | 1.37E-01 | TRH;NTS;APLN |
| G Alpha (Q) Signaling Events R-HSA-416476 | 3/212 | 1.60E-02 | 1.55E-01 | GNG5;TRH;NTS |
| Fcgamma Receptor (FCGR) Dependent Phagocytosis R-HSA-2029480 | 2/87 | 2.00E-02 | 1.55E-01 | ACTR2;ARPC3 |
| Chaperonin-mediated Protein Folding R-HSA-390466 | 2/90 | 2.13E-02 | 1.55E-01 | TUBA1C;GNG5 |
| Signaling By Rho GTPases R-HSA-194315 | 5/644 | 2.19E-02 | 1.55E-01 | ACTR2;YWHAB;ARPC3;VIM;MYL12B |
| Class B/2 (Secretin Family Receptors) R-HSA-373080 | 2/92 | 2.22E-02 | 1.55E-01 | RAMP2;GNG5 |
| Protein Folding R-HSA-391251 | 2/96 | 2.41E-02 | 1.55E-01 | TUBA1C;GNG5 |
| Signaling By Rho GTPases, Miro GTPases And RHOBTB3 R-HSA-9716542 | 5/660 | 2.41E-02 | 1.55E-01 | ACTR2;YWHAB;ARPC3;VIM;MYL12B |
| Neutrophil Degranulation R-HSA-6798695 | 4/468 | 2.93E-02 | 1.55E-01 | ACTR2;DYNLT1;XRCC5;PDAP1 |
| Infectious Disease R-HSA-5663205 | 6/961 | 3.18E-02 | 1.55E-01 | ACTR2;RAMP2;GNG5;YWHAB;XRCC5;ARPC3 |
| Translation R-HSA-72766 | 3/281 | 3.31E-02 | 1.55E-01 | SPCS3;EIF4EBP1;EIF2S2 |
| Cap-dependent Translation Initiation R-HSA-72737 | 2/116 | 3.41E-02 | 1.55E-01 | EIF4EBP1;EIF2S2 |
| Axon Guidance R-HSA-422475 | 4/519 | 4.04E-02 | 1.55E-01 | ACTR2;ARPC3;DPYSL2;MYL12B |
| ADORA2B Mediated Anti-Inflammatory Cytokine Production R-HSA-9660821 | 2/131 | 4.25E-02 | 1.55E-01 | RAMP2;GNG5 |
| G Alpha (I) Signaling Events R-HSA-418594 | 3/312 | 4.30E-02 | 1.55E-01 | GNG5;RGS10;APLN |
| Innate Immune System R-HSA-168249 | 6/1035 | 4.33E-02 | 1.55E-01 | ACTR2;DYNLT1;CD81;XRCC5;ARPC3;PDAP1 |
| Nervous System Development R-HSA-9675108 | 4/545 | 4.69E-02 | 1.55E-01 | ACTR2;ARPC3;DPYSL2;MYL12B |
| Class A/1 (Rhodopsin-like Receptors) R-HSA-373076 | 3/327 | 4.83E-02 | 1.55E-01 | TRH;NTS;APLN |
| Clathrin-mediated Endocytosis R-HSA-8856828 | 2/142 | 4.91E-02 | 1.55E-01 | ACTR2;ARPC3 |


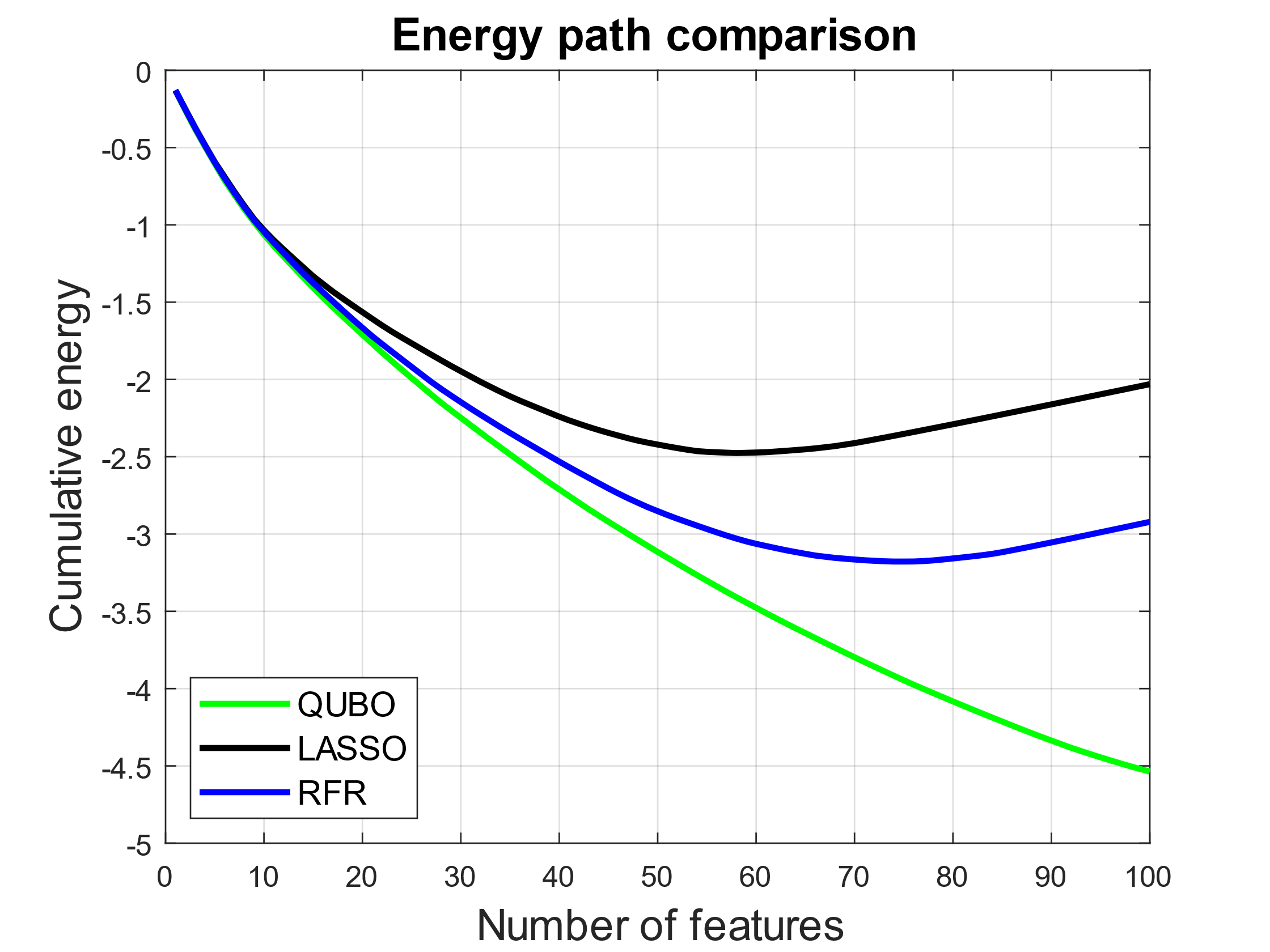


**Supplementary Fig. S1. Energy path feature selection (100) of smaller search space.** Energy path comparison of QUBO against RFR and LASSO feature selection for the top 100 features. The energy path represents the cumulative energy contribution of sequentially selected features, akin to climbing a ladder where each step represents a feature’s energy score. This visualization illustrates the ability of the QUBO cost function to effectively minimize energy, leading to the selection of unique feature combinations and avoiding co-regulated features, thereby facilitating the identification of master regulators. RFR and LASSO tend to select redundant or co-regulated features, making the curve to go up.

**Supplementary Table 2. Enrichr Pathways of QUBO-Selected Genes in the Drug Resistance Dataset.** Significant Enrichr pathway analysis results for genes selected by the QUBO method in the drug resistance dataset are shown below.

| Term | Overlap | P-value | Adj.  P-value | Genes |
| --- | --- | --- | --- | --- |
| Regulation Of Apoptotic Process (GO:0042981) | 18/705 | 1.17E-08 | 1.32E-05 | TOP2A;HSP90AA1;ANXA1;CITED2;IGFBP3;WNT5A;FSTL1;CFL1;TAX1BP1;CYP1B1;BIRC5;ITGA6;ANP32B;SQSTM1;PPIA;PTMA;CD44;TGM2 |
| Epithelial Cell Differentiation (GO:0030855) | 8/132 | 3.25E-07 | 1.85E-04 | LGALS3;GSTK1;KRT19;ANXA1;PCNA;KRT17;KRT13;FSTL1 |
| Positive Regulation By Host Of Viral Process (GO:0044794) | 4/17 | 1.33E-06 | 5.04E-04 | EEF1A1;KRT17;CFL1;NUCKS1 |
| Epithelial Cell Migration (GO:0010631) | 4/46 | 8.17E-05 | 2.32E-02 | ANLN;S100A2;CYP1B1;FSTL1 |
| Epithelium Development (GO:0060429) | 6/154 | 1.24E-04 | 2.35E-02 | LGALS3;GSTK1;KRT19;PCNA;KRT17;KRT13 |
| Negative Regulation Of Programmed Cell Death (GO:0043069) | 9/381 | 1.24E-04 | 2.35E-02 | ANXA1;CITED2;CFL1;WNT5A;TAX1BP1;BIRC5;PTMA;FSTL1;CD44 |
| Modulation By Host Of Viral Process (GO:0044788) | 3/22 | 1.74E-04 | 2.44E-02 | KRT17;CFL1;NUCKS1 |
| Supramolecular Fiber Organization (GO:0097435) | 8/316 | 1.88E-04 | 2.44E-02 | KRT19;KRT17;TPM3;STMN1;TPM1;KRT13;CYP1B1;ALDOA |
| Mitotic Cytokinesis (GO:0000281) | 4/59 | 2.17E-04 | 2.44E-02 | ANLN;CFL1;STMN1;BIRC5 |
| protein-DNA Complex Organization (GO:0071824) | 4/60 | 2.31E-04 | 2.44E-02 | CBX3;HMGB2;NUCKS1;HMGN2 |
| Glycolipid Transport (GO:0046836) | 2/5 | 2.45E-04 | 2.44E-02 | NPC2;PSAP |
| Negative Regulation Of Stress Fiber Assembly (GO:0051497) | 3/25 | 2.58E-04 | 2.44E-02 | STMN1;TACSTD2;PFN1 |
| Positive Regulation Of DNA Biosynthetic Process (GO:2000573) | 4/65 | 3.15E-04 | 2.60E-02 | CCT6A;HSP90AA1;PCNA;USP1 |
| Negative Regulation Of Actin Filament Bundle Assembly (GO:0032232) | 3/28 | 3.63E-04 | 2.60E-02 | STMN1;TACSTD2;PFN1 |
| Hepatocyte Growth Factor Receptor Signaling Pathway (GO:0048012) | 2/6 | 3.66E-04 | 2.60E-02 | STMN1;MUC20 |
| Intracellular Sequestering Of Iron Ion (GO:0006880) | 2/6 | 3.66E-04 | 2.60E-02 | FTH1;FTL |
| Chromatin Organization (GO:0006325) | 7/268 | 3.98E-04 | 2.64E-02 | NASP;CBX3;HMGB2;NUCKS1;ANP32B;DEK;HMGN2 |
| Negative Regulation Of Supramolecular Fiber Organization (GO:1902904) | 4/71 | 4.42E-04 | 2.64E-02 | LIMA1;TPX2;STMN1;PFN1 |
| Cellular Response To Reactive Oxygen Species (GO:0034614) | 4/73 | 4.92E-04 | 2.64E-02 | ANXA1;TPM1;CYP1B1;SQSTM1 |
| Aromatic Amino Acid Transport (GO:0015801) | 2/7 | 5.11E-04 | 2.64E-02 | SLC7A5;SLC3A2 |
| Sulfur Amino Acid Transport (GO:0000101) | 2/7 | 5.11E-04 | 2.64E-02 | SLC7A5;SLC3A2 |
| Thyroid Hormone Transport (GO:0070327) | 2/7 | 5.11E-04 | 2.64E-02 | SLC7A5;SLC3A2 |
| Regulation Of Stress Fiber Assembly (GO:0051492) | 4/75 | 5.45E-04 | 2.69E-02 | STMN1;TPM1;TACSTD2;PFN1 |
| Response To Estrogen (GO:0043627) | 3/34 | 6.48E-04 | 2.93E-02 | KRT19;CITED2;CD24 |
| DNA Topological Change (GO:0006265) | 2/8 | 6.80E-04 | 2.93E-02 | TOP2A;HMGB2 |
| Proline Transport (GO:0015824) | 2/8 | 6.80E-04 | 2.93E-02 | SLC7A5;SLC3A2 |
| Negative Regulation Of Apoptotic Process (GO:0043066) | 9/482 | 6.96E-04 | 2.93E-02 | ANXA1;CITED2;CFL1;WNT5A;TAX1BP1;BIRC5;PTMA;FSTL1;CD44 |
| Cytoskeleton-Dependent Cytokinesis (GO:0061640) | 4/81 | 7.29E-04 | 2.95E-02 | ANLN;CFL1;STMN1;BIRC5 |
| Positive Regulation Of ATP Metabolic Process (GO:1903580) | 2/9 | 8.71E-04 | 3.19E-02 | TMSB4X;ENO1 |
| Hormone Transport (GO:0009914) | 2/9 | 8.71E-04 | 3.19E-02 | SLC7A5;SLC3A2 |
| Leucine Transport (GO:0015820) | 2/9 | 8.71E-04 | 3.19E-02 | SLC7A5;SLC3A2 |
| Positive Regulation Of Cell Adhesion (GO:0045785) | 4/88 | 9.95E-04 | 3.53E-02 | CCDC80;CITED2;TPM1;TGM2 |
| Endothelial Cell Migration (GO:0043542) | 3/40 | 1.05E-03 | 3.60E-02 | S100A2;CYP1B1;FSTL1 |
| Branched-Chain Amino Acid Transport (GO:0015803) | 2/10 | 1.09E-03 | 3.62E-02 | SLC7A5;SLC3A2 |
| Negative Regulation Of Cell Migration (GO:0030336) | 5/163 | 1.37E-03 | 4.43E-02 | CITED2;IGFBP3;TPM1;TACSTD2;CYP1B1 |
| Positive Regulation Of Programmed Cell Death (GO:0043068) | 6/245 | 1.45E-03 | 4.58E-02 | TOP2A;IGFBP3;CYP1B1;ITGA6;SQSTM1;TGM2 |
| Regulation Of Fibroblast Proliferation (GO:0048145) | 3/46 | 1.58E-03 | 4.72E-02 | FTH1;WNT5A;S100A6 |
| DNA Conformation Change (GO:0071103) | 2/12 | 1.58E-03 | 4.72E-02 | TOP2A;HMGB2 |
| Positive Regulation Of Chromosome Separation (GO:1905820) | 2/13 | 1.86E-03 | 5.30E-02 | BIRC5;SMC4 |
| Unsaturated Fatty Acid Metabolic Process (GO:0033559) | 3/49 | 1.89E-03 | 5.30E-02 | SCD;CYP1B1;PTGR1 |
| Positive Regulation Of Hydrolase Activity (GO:0051345) | 5/176 | 1.91E-03 | 5.30E-02 | PSAP;HMGB2;ITGA6;CALM2;TGM2 |
| Negative Regulation Of Nucleic Acid-Templated Transcription (GO:1903507) | 8/456 | 2.05E-03 | 5.54E-02 | ELF3;CBX3;CITED2;WNT5A;HMGA1;HMGB2;BIRC5;ENO1 |
| Negative Regulation Of Smooth Muscle Cell Migration (GO:0014912) | 2/14 | 2.17E-03 | 5.59E-02 | IGFBP3;TPM1 |
| Regulation Of ATP Biosynthetic Process (GO:2001169) | 2/14 | 2.17E-03 | 5.59E-02 | TMSB4X;ENO1 |
| Positive Regulation Of Apoptotic Process (GO:0043065) | 6/270 | 2.37E-03 | 5.85E-02 | TOP2A;IGFBP3;CYP1B1;ITGA6;SQSTM1;TGM2 |
| Carboxylic Acid Transport (GO:0046942) | 3/53 | 2.37E-03 | 5.85E-02 | SLC7A5;PSAP;SLC3A2 |
| Nitric Oxide Biosynthetic Process (GO:0006809) | 2/15 | 2.49E-03 | 6.02E-02 | NQO1;CYP1B1 |
| Cellular Response To Oxidative Stress (GO:0034599) | 4/117 | 2.83E-03 | 6.08E-02 | NQO1;TPM1;SQSTM1;PPIA |
| Positive Regulation Of Protein Serine/Threonine Kinase Activity (GO:0071902) | 4/117 | 2.83E-03 | 6.08E-02 | CCND1;WNT5A;CD24;CALM2 |
| Nitric Oxide Metabolic Process (GO:0046209) | 2/16 | 2.84E-03 | 6.08E-02 | NQO1;CYP1B1 |
| Regulation Of Response To Cytokine Stimulus (GO:0060759) | 2/16 | 2.84E-03 | 6.08E-02 | WNT5A;CD24 |
| Retinal Metabolic Process (GO:0042574) | 2/16 | 2.84E-03 | 6.08E-02 | ALDH1A3;CYP1B1 |
| Ruffle Organization (GO:0031529) | 2/16 | 2.84E-03 | 6.08E-02 | LIMA1;TPM1 |
| Regulation Of Protein Catabolic Process (GO:0042176) | 4/122 | 3.29E-03 | 6.92E-02 | EEF1A1;NQO1;HSP90AA1;WNT5A |
| Negative Regulation Of Intrinsic Apoptotic Signaling Pathway (GO:2001243) | 3/61 | 3.54E-03 | 7.30E-02 | ENO1;YBX3;PPIA |
| Negative Regulation Of Cell Motility (GO:2000146) | 4/133 | 4.48E-03 | 9.08E-02 | CITED2;TPM1;TACSTD2;CYP1B1 |
| Skin Development (GO:0043588) | 3/68 | 4.80E-03 | 9.40E-02 | ANXA1;WNT5A;ITGA6 |
| Intermediate Filament Organization (GO:0045109) | 3/68 | 4.80E-03 | 9.40E-02 | KRT19;KRT17;KRT13 |
| Modulation By Host Of Viral Genome Replication (GO:0044827) | 2/21 | 4.89E-03 | 9.40E-02 | EEF1A1;NUCKS1 |
| Cytoskeleton-Dependent Intracellular Transport (GO:0030705) | 2/22 | 5.36E-03 | 9.92E-02 | TUBA1C;TUBA1B |
| Positive Regulation Of Protein Modification Process (GO:0031401) | 5/225 | 5.46E-03 | 9.92E-02 | HSP90AA1;CCND1;BIRC5;PPIA;CALM2 |
| Cholesterol Homeostasis (GO:0042632) | 3/72 | 5.64E-03 | 9.92E-02 | LIMA1;NPC2;CD24 |
| Negative Regulation Of Protein Depolymerization (GO:1901880) | 2/23 | 5.85E-03 | 9.92E-02 | LIMA1;TPX2 |
| Cell-Cell Signaling By Wnt (GO:0198738) | 2/23 | 5.85E-03 | 9.92E-02 | WNT5A;CD24 |
| Negative Regulation Of Actin Filament Polymerization (GO:0030837) | 2/23 | 5.85E-03 | 9.92E-02 | TMSB4X;PFN1 |
| Sterol Homeostasis (GO:0055092) | 3/73 | 5.86E-03 | 9.92E-02 | LIMA1;NPC2;CD24 |
| Granulocyte Chemotaxis (GO:0071621) | 3/73 | 5.86E-03 | 9.92E-02 | LGALS3;ANXA1;PPIA |
| Positive Regulation Of Phosphorylation (GO:0042327) | 5/231 | 6.10E-03 | 1.00E-01 | HSP90AA1;CCND1;BIRC5;ITGA6;PPIA |
| Nucleosome Organization (GO:0034728) | 3/75 | 6.31E-03 | 1.00E-01 | NASP;HMGB2;HMGA1 |
| Regulation Of Cytokine Production Involved In Immune Response (GO:0002718) | 2/24 | 6.36E-03 | 1.00E-01 | SLC7A5;WNT5A |
| Regulation Of Ruffle Assembly (GO:1900027) | 2/24 | 6.36E-03 | 1.00E-01 | TACSTD2;PFN1 |
| Negative Regulation Of Protein Metabolic Process (GO:0051248) | 4/147 | 6.37E-03 | 1.00E-01 | CSTB;NQO1;NCL;TYMS |
| Negative Regulation Of Protein Phosphorylation (GO:0001933) | 4/149 | 6.67E-03 | 1.03E-01 | IGFBP3;PMEPA1;PPIA;CALM2 |
| Actomyosin Structure Organization (GO:0031032) | 3/77 | 6.79E-03 | 1.03E-01 | PDLIM1;KRT19;TPM1 |
| Activation Of Protein Kinase B Activity (GO:0032148) | 2/25 | 6.89E-03 | 1.03E-01 | WNT5A;PPIA |
| Regulation Of Postsynapse Organization (GO:0099175) | 2/25 | 6.89E-03 | 1.03E-01 | CFL1;WNT5A |
| Long-Chain Fatty Acid Metabolic Process (GO:0001676) | 3/78 | 7.04E-03 | 1.04E-01 | CYP1B1;ACSL3;PTGR1 |
| L-amino Acid Transport (GO:0015807) | 2/26 | 7.44E-03 | 1.07E-01 | SLC7A5;SLC3A2 |
| Negative Regulation Of Protein Polymerization (GO:0032272) | 2/26 | 7.44E-03 | 1.07E-01 | STMN1;PFN1 |
| Cellular Response To Growth Factor Stimulus (GO:0071363) | 4/155 | 7.65E-03 | 1.07E-01 | EEF1A1;ANXA1;WNT5A;CD44 |
| Activation Of Protein Kinase Activity (GO:0032147) | 3/81 | 7.81E-03 | 1.07E-01 | TPX2;WNT5A;PPIA |
| Negative Regulation Of NF-kappaB Transcription Factor Activity (GO:0032088) | 3/81 | 7.81E-03 | 1.07E-01 | TMSB4X;TAX1BP1;CYP1B1 |
| Amino Acid Import Across Plasma Membrane (GO:0089718) | 2/27 | 8.01E-03 | 1.07E-01 | SLC7A5;SLC3A2 |
| Peptide Cross-Linking (GO:0018149) | 2/27 | 8.01E-03 | 1.07E-01 | ANXA1;TGM2 |
| Cell Surface Receptor Signaling Pathway Involved In Cell-Cell Signaling (GO:1905114) | 2/27 | 8.01E-03 | 1.07E-01 | WNT5A;CD24 |
| Apoptotic Cell Clearance (GO:0043277) | 2/28 | 8.60E-03 | 1.11E-01 | ANXA1;TGM2 |
| Positive Regulation Of Fibroblast Proliferation (GO:0048146) | 2/28 | 8.60E-03 | 1.11E-01 | WNT5A;S100A6 |
| Negative Regulation Of Cytoskeleton Organization (GO:0051494) | 2/28 | 8.60E-03 | 1.11E-01 | LIMA1;PFN1 |
| Mitotic Spindle Organization (GO:0007052) | 3/85 | 8.91E-03 | 1.12E-01 | TPX2;STMN1;BIRC5 |
| Regulation Of Muscle Contraction (GO:0006937) | 2/29 | 9.20E-03 | 1.12E-01 | TPM1;ENO1 |
| Sarcomere Organization (GO:0045214) | 2/29 | 9.20E-03 | 1.12E-01 | KRT19;TPM1 |
| Positive Regulation Of Dephosphorylation (GO:0035306) | 2/29 | 9.20E-03 | 1.12E-01 | CALM2;PPIA |
| Positive Regulation Of Viral Genome Replication (GO:0045070) | 2/29 | 9.20E-03 | 1.12E-01 | TOP2A;PPIA |
| DNA Geometric Change (GO:0032392) | 2/30 | 9.83E-03 | 1.19E-01 | HNRNPA2B1;HMGB2 |
| Negative Regulation Of Microtubule Polymerization Or Depolymerization (GO:0031111) | 2/31 | 1.05E-02 | 1.23E-01 | TPX2;STMN1 |
| Regulation Of Long-Term Synaptic Potentiation (GO:1900271) | 2/31 | 1.05E-02 | 1.23E-01 | CALB2;SQSTM1 |
| Regulation Of Cell Morphogenesis (GO:0022604) | 2/31 | 1.05E-02 | 1.23E-01 | CFL1;CD44 |
| Regulation Of Protein Phosphorylation (GO:0001932) | 5/265 | 1.07E-02 | 1.24E-01 | HSP90AA1;CCND1;IGFBP3;BIRC5;PPIA |
| Cellular Nitrogen Compound Biosynthetic Process (GO:0044271) | 2/32 | 1.11E-02 | 1.25E-01 | NQO1;CYP1B1 |
| Chromosome Condensation (GO:0030261) | 2/32 | 1.11E-02 | 1.25E-01 | TOP2A;SMC4 |
| Gluconeogenesis (GO:0006094) | 2/32 | 1.11E-02 | 1.25E-01 | KRT17;ENO1 |
| Positive Regulation Of Protein Phosphorylation (GO:0001934) | 6/377 | 1.17E-02 | 1.27E-01 | HSP90AA1;CCND1;BIRC5;PPIA;CALM2;CD44 |
| Muscle Contraction (GO:0006936) | 3/94 | 1.17E-02 | 1.27E-01 | TPM3;TPM1;ALDOA |
| Regulation Of Protein Polymerization (GO:0032271) | 2/33 | 1.18E-02 | 1.27E-01 | HSP90AA1;PFN1 |
| Positive Regulation Of Cytokine Production Involved In Immune Response (GO:0002720) | 2/33 | 1.18E-02 | 1.27E-01 | SLC7A5;WNT5A |
| Positive Regulation Of Protein Dephosphorylation (GO:0035307) | 2/33 | 1.18E-02 | 1.27E-01 | CALM2;PPIA |
| Cellular Response To Hydrogen Peroxide (GO:0070301) | 2/34 | 1.25E-02 | 1.30E-01 | ANXA1;CYP1B1 |
| Sister Chromatid Segregation (GO:0000819) | 2/34 | 1.25E-02 | 1.30E-01 | TOP2A;SMC4 |
| Positive Regulation Of Transcription By RNA Polymerase I (GO:0045943) | 2/34 | 1.25E-02 | 1.30E-01 | NCL;DEK |
| Regulation Of Defense Response (GO:0031347) | 3/97 | 1.27E-02 | 1.31E-01 | ANXA1;TMSB4X;WNT5A |
| Neutral Amino Acid Transport (GO:0015804) | 2/35 | 1.32E-02 | 1.33E-01 | SLC7A5;SLC3A2 |
| Hexose Biosynthetic Process (GO:0019319) | 2/35 | 1.32E-02 | 1.33E-01 | KRT17;ENO1 |
| Positive Regulation Of Telomere Maintenance Via Telomere Lengthening (GO:1904358) | 2/35 | 1.32E-02 | 1.33E-01 | CCT6A;HNRNPA2B1 |
| Response To UV (GO:0009411) | 3/100 | 1.38E-02 | 1.38E-01 | PCNA;CCND1;USP1 |
| Negative Regulation Of Smooth Muscle Cell Proliferation (GO:0048662) | 2/37 | 1.47E-02 | 1.45E-01 | IGFBP3;TPM1 |
| Positive Regulation Of Cation Channel Activity (GO:2001259) | 2/38 | 1.55E-02 | 1.50E-01 | TMSB4X;CALM2 |
| Positive Regulation Of Production Of Molecular Mediator Of Immune Response (GO:0002702) | 2/38 | 1.55E-02 | 1.50E-01 | SLC7A5;WNT5A |
| Intrinsic Apoptotic Signaling Pathway (GO:0097193) | 3/105 | 1.57E-02 | 1.51E-01 | CYP1B1;CYCS;CD24 |
| Regulation Of Signal Transduction (GO:0009966) | 4/193 | 1.61E-02 | 1.53E-01 | IGFBP3;NUCKS1;CD24;PPIA |
| Positive Regulation Of ATP-dependent Activity (GO:0032781) | 2/39 | 1.63E-02 | 1.54E-01 | TPM1;PFN1 |
| Regulation Of Intracellular Signal Transduction (GO:1902531) | 5/297 | 1.67E-02 | 1.55E-01 | TMSB4X;WNT5A;CD24;SQSTM1;CALM2 |
| Regulation Of Cytosolic Calcium Ion Concentration (GO:0051480) | 2/40 | 1.71E-02 | 1.55E-01 | CALB2;WNT5A |
| L-alpha-amino Acid Transmembrane Transport (GO:1902475) | 2/40 | 1.71E-02 | 1.55E-01 | SLC7A5;SLC3A2 |
| Regulation Of Protein Dephosphorylation (GO:0035304) | 2/40 | 1.71E-02 | 1.55E-01 | CALM2;PPIA |
| Retinoid Metabolic Process (GO:0001523) | 2/40 | 1.71E-02 | 1.55E-01 | ALDH1A3;CYP1B1 |
| Cell-Matrix Adhesion (GO:0007160) | 3/109 | 1.74E-02 | 1.57E-01 | ITGA2;ITGA6;CD44 |
| Response To Lipid (GO:0033993) | 3/110 | 1.78E-02 | 1.59E-01 | ANXA1;SCD;HMGB2 |
| Negative Regulation Of Cellular Process (GO:0048523) | 7/537 | 1.82E-02 | 1.60E-01 | FTH1;IGFBP3;TACSTD2;HMGA1;CYP1B1;ENO1;ANP32B |
| Cytoskeleton Organization (GO:0007010) | 3/111 | 1.82E-02 | 1.60E-01 | CFL1;TPM1;KRT13 |
| Regulation Of Translation (GO:0006417) | 4/202 | 1.87E-02 | 1.62E-01 | EIF5B;NCL;KRT13;TYMS |
| Keratinocyte Differentiation (GO:0030216) | 2/42 | 1.87E-02 | 1.62E-01 | ANXA1;WNT5A |
| Positive Regulation Of Transcription By RNA Polymerase II (GO:0045944) | 10/938 | 1.89E-02 | 1.62E-01 | ELF3;CITED2;WNT5A;HMGA1;HMGB2;NUCKS1;ITGA6;DEK;SQSTM1;PTMA |
| DNA-templated DNA Replication Maintenance Of Fidelity (GO:0045005) | 2/43 | 1.96E-02 | 1.67E-01 | PCNA;NUCKS1 |
| Mitotic Spindle Assembly (GO:0090307) | 2/44 | 2.04E-02 | 1.73E-01 | TPX2;BIRC5 |
| Regulation Of Cellular Metabolic Process (GO:0031323) | 2/45 | 2.13E-02 | 1.75E-01 | NPC2;CYP1B1 |
| Regulation Of Cell Migration (GO:0030334) | 6/434 | 2.18E-02 | 1.75E-01 | ANXA1;CITED2;TMSB4X;TPM1;CYP1B1;ITGA6 |
| Amino Acid Transport (GO:0006865) | 2/46 | 2.22E-02 | 1.75E-01 | SLC7A5;SLC3A2 |
| Replication Fork Processing (GO:0031297) | 2/46 | 2.22E-02 | 1.75E-01 | PCNA;NUCKS1 |
| Positive Regulation Of Blood Vessel Endothelial Cell Migration (GO:0043536) | 2/46 | 2.22E-02 | 1.75E-01 | ANXA1;TMSB4X |
| Response To Hydrogen Peroxide (GO:0042542) | 2/46 | 2.22E-02 | 1.75E-01 | ANXA1;CYP1B1 |
| Positive Regulation Of Cell-Cell Adhesion (GO:0022409) | 2/46 | 2.22E-02 | 1.75E-01 | CITED2;CD44 |
| Myofibril Assembly (GO:0030239) | 2/46 | 2.22E-02 | 1.75E-01 | KRT19;TPM1 |
| Epithelial To Mesenchymal Transition (GO:0001837) | 2/47 | 2.31E-02 | 1.75E-01 | WNT5A;S100A4 |
| Negative Regulation Of Endoplasmic Reticulum Calcium Ion Concentration (GO:0032471) | 1/5 | 2.48E-02 | 1.75E-01 | TGM2 |
| DNA Replication-Dependent Chromatin Assembly (GO:0006335) | 1/5 | 2.48E-02 | 1.75E-01 | NASP |
| Negative Regulation Of Morphogenesis Of An Epithelium (GO:1905331) | 1/5 | 2.48E-02 | 1.75E-01 | TACSTD2 |
| Negative Regulation Of Protein Polyubiquitination (GO:1902915) | 1/5 | 2.48E-02 | 1.75E-01 | PPIA |
| Activation Of Cysteine-Type Endopeptidase Activity Involved In Apoptotic Process By Cytochrome C (GO:0008635) | 1/5 | 2.48E-02 | 1.75E-01 | CYCS |
| Negative Regulation Of Transcription From RNA Polymerase II Promoter In Response To Stress (GO:0097201) | 1/5 | 2.48E-02 | 1.75E-01 | CITED2 |
| Pexophagy (GO:0000425) | 1/5 | 2.48E-02 | 1.75E-01 | SQSTM1 |
| Renal Filtration Cell Differentiation (GO:0061318) | 1/5 | 2.48E-02 | 1.75E-01 | CD24 |
| Glomerular Epithelial Cell Differentiation (GO:0072311) | 1/5 | 2.48E-02 | 1.75E-01 | CD24 |
| Positive Regulation Of Mitotic Cytokinesis (GO:1903490) | 1/5 | 2.48E-02 | 1.75E-01 | BIRC5 |
| Positive Regulation Of Peroxisome Proliferator Activated Receptor Signaling Pathway (GO:0035360) | 1/5 | 2.48E-02 | 1.75E-01 | CITED2 |
| Positive Regulation Of Tau-Protein Kinase Activity (GO:1902949) | 1/5 | 2.48E-02 | 1.75E-01 | HSP90AA1 |
| Iron Coordination Entity Transport (GO:1901678) | 1/5 | 2.48E-02 | 1.75E-01 | LCN2 |
| Positive Regulation Of Vesicle Fusion (GO:0031340) | 1/5 | 2.48E-02 | 1.75E-01 | ANXA1 |
| Protein Targeting To Vacuole Involved In Autophagy (GO:0071211) | 1/5 | 2.48E-02 | 1.75E-01 | SQSTM1 |
| Regulation Of T Cell Activation Via T Cell Receptor Contact With Antigen Bound To MHC Molecule On Antigen Presenting Cell (GO:2001188) | 1/5 | 2.48E-02 | 1.75E-01 | LGALS3 |
| Negative Regulation Of Actin Filament Depolymerization (GO:0030835) | 1/5 | 2.48E-02 | 1.75E-01 | LIMA1 |
| Negative Regulation Of Cellular Response To Hypoxia (GO:1900038) | 1/5 | 2.48E-02 | 1.75E-01 | ENO1 |
| Regulation Of Response To External Stimulus (GO:0032101) | 3/127 | 2.59E-02 | 1.76E-01 | ANXA1;TMSB4X;WNT5A |
| Regulation Of Epithelial Cell Migration (GO:0010632) | 2/50 | 2.59E-02 | 1.76E-01 | TACSTD2;PFN1 |
| Intracellular Iron Ion Homeostasis (GO:0006879) | 2/50 | 2.59E-02 | 1.76E-01 | FTH1;FTL |
| Pyruvate Metabolic Process (GO:0006090) | 2/50 | 2.59E-02 | 1.76E-01 | KRT17;ENO1 |
| Negative Regulation Of Gene Expression (GO:0010629) | 5/336 | 2.68E-02 | 1.76E-01 | SLC7A5;KRT17;CITED2;NCL;TYMS |
| Cholesterol Transport (GO:0030301) | 2/51 | 2.69E-02 | 1.76E-01 | LIMA1;NPC2 |
| Positive Regulation Of Actin Filament Bundle Assembly (GO:0032233) | 2/51 | 2.69E-02 | 1.76E-01 | TPM1;PFN1 |
| Positive Regulation Of Protein Localization To Cell Periphery (GO:1904377) | 2/51 | 2.69E-02 | 1.76E-01 | LGALS3;SQSTM1 |
| Positive Regulation Of Protein Localization To Plasma Membrane (GO:1903078) | 2/51 | 2.69E-02 | 1.76E-01 | LGALS3;SQSTM1 |
| Regulation Of DNA Repair (GO:0006282) | 3/129 | 2.69E-02 | 1.76E-01 | PCNA;USP1;DEK |
| Regulation Of Proteolysis (GO:0030162) | 2/52 | 2.79E-02 | 1.76E-01 | CSTB;RHOBTB3 |
| Icosanoid Metabolic Process (GO:0006690) | 2/52 | 2.79E-02 | 1.76E-01 | CYP1B1;PTGR1 |
| Plasma Membrane Bounded Cell Projection Organization (GO:0120036) | 3/132 | 2.86E-02 | 1.76E-01 | LIMA1;STMN1;TPM1 |
| Protein Homotetramerization (GO:0051289) | 2/53 | 2.89E-02 | 1.76E-01 | ALDH1A3;ALDOA |
| CENP-A Containing Chromatin Assembly (GO:0034080) | 1/6 | 2.96E-02 | 1.76E-01 | NASP |
| G-quadruplex DNA Unwinding (GO:0044806) | 1/6 | 2.96E-02 | 1.76E-01 | HNRNPA2B1 |
| Negative Regulation Of Ruffle Assembly (GO:1900028) | 1/6 | 2.96E-02 | 1.76E-01 | TACSTD2 |
| Aggrephagy (GO:0035973) | 1/6 | 2.96E-02 | 1.76E-01 | SQSTM1 |
| Negative Regulation Of Stress-Activated Protein Kinase Signaling Cascade (GO:0070303) | 1/6 | 2.96E-02 | 1.76E-01 | PPIA |
| Regulation Of Mitotic Cytokinesis (GO:1902412) | 1/6 | 2.96E-02 | 1.76E-01 | BIRC5 |
| Bleb Assembly (GO:0032060) | 1/6 | 2.96E-02 | 1.76E-01 | EMP1 |
| Cellular Hyperosmotic Response (GO:0071474) | 1/6 | 2.96E-02 | 1.76E-01 | YBX3 |
| Cellular Response To Monoamine Stimulus (GO:0071868) | 1/6 | 2.96E-02 | 1.76E-01 | TGM2 |
| Chromatin Remodeling At Centromere (GO:0031055) | 1/6 | 2.96E-02 | 1.76E-01 | NASP |
| Response To Cocaine (GO:0042220) | 1/6 | 2.96E-02 | 1.76E-01 | TGM2 |
| Positive Regulation Of Unsaturated Fatty Acid Biosynthetic Process (GO:2001280) | 1/6 | 2.96E-02 | 1.76E-01 | ANXA1 |
| Macrophage Derived Foam Cell Differentiation (GO:0010742) | 1/6 | 2.96E-02 | 1.76E-01 | WNT5A |
| Regulation Of Lipid Metabolic Process (GO:0019216) | 2/54 | 2.99E-02 | 1.76E-01 | NPC2;PSAP |
| Nucleosome Assembly (GO:0006334) | 2/54 | 2.99E-02 | 1.76E-01 | NASP;HMGB2 |
| Epidermal Cell Differentiation (GO:0009913) | 2/54 | 2.99E-02 | 1.76E-01 | ANXA1;WNT5A |
| Mesenchymal Cell Differentiation (GO:0048762) | 2/54 | 2.99E-02 | 1.76E-01 | WNT5A;S100A4 |
| Inflammatory Response (GO:0006954) | 4/236 | 3.08E-02 | 1.76E-01 | ANXA1;ELF3;WNT5A;CD44 |
| Monoatomic Anion Transport (GO:0006820) | 2/57 | 3.31E-02 | 1.76E-01 | FXYD3;BEST1 |
| Response To Organic Cyclic Compound (GO:0014070) | 2/58 | 3.41E-02 | 1.76E-01 | ANXA1;CYP1B1 |
| Inorganic Anion Transport (GO:0015698) | 2/58 | 3.41E-02 | 1.76E-01 | FXYD3;BEST1 |
| Regulation Of Chaperone-Mediated Autophagy (GO:1904714) | 1/7 | 3.45E-02 | 1.76E-01 | EEF1A1 |
| Regulation Of Chromosome Condensation (GO:0060623) | 1/7 | 3.45E-02 | 1.76E-01 | SMC4 |
| Regulation Of Cyclic-Nucleotide Phosphodiesterase Activity (GO:0051342) | 1/7 | 3.45E-02 | 1.76E-01 | CALM2 |
| DNA Ligation (GO:0006266) | 1/7 | 3.45E-02 | 1.76E-01 | TOP2A |
| Negative Regulation Of Neural Precursor Cell Proliferation (GO:2000178) | 1/7 | 3.45E-02 | 1.76E-01 | WNT5A |
| Negative Regulation Of Phospholipase Activity (GO:0010519) | 1/7 | 3.45E-02 | 1.76E-01 | ANXA1 |
| Regulation Of Lymphocyte Apoptotic Process (GO:0070228) | 1/7 | 3.45E-02 | 1.76E-01 | LGALS3 |
| Regulation Of Phospholipase A2 Activity (GO:0032429) | 1/7 | 3.45E-02 | 1.76E-01 | ANXA1 |
| Positive Regulation By Host Of Viral Genome Replication (GO:0044829) | 1/7 | 3.45E-02 | 1.76E-01 | NUCKS1 |
| Positive Regulation Of Attachment Of Mitotic Spindle Microtubules To Kinetochore (GO:1902425) | 1/7 | 3.45E-02 | 1.76E-01 | BIRC5 |
| Cytoplasmic Sequestering Of NF-kappaB (GO:0007253) | 1/7 | 3.45E-02 | 1.76E-01 | TMSB4X |
| Positive Regulation Of Calcium Ion Import (GO:0090280) | 1/7 | 3.45E-02 | 1.76E-01 | LGALS3 |
| Positive Regulation Of Chromosome Condensation (GO:1905821) | 1/7 | 3.45E-02 | 1.76E-01 | SMC4 |
| Spleen Development (GO:0048536) | 1/7 | 3.45E-02 | 1.76E-01 | CITED2 |
| Positive Regulation Of Leukocyte Differentiation (GO:1902107) | 1/7 | 3.45E-02 | 1.76E-01 | LGALS3 |
| Foam Cell Differentiation (GO:0090077) | 1/7 | 3.45E-02 | 1.76E-01 | WNT5A |
| Fructose Metabolic Process (GO:0006000) | 1/7 | 3.45E-02 | 1.76E-01 | ALDOA |
| Positive Regulation Of Myeloid Cell Apoptotic Process (GO:0033034) | 1/7 | 3.45E-02 | 1.76E-01 | ANXA1 |
| Positive Regulation Of Neuron Projection Arborization (GO:0150012) | 1/7 | 3.45E-02 | 1.76E-01 | WNT5A |
| Positive Regulation Of Plasminogen Activation (GO:0010756) | 1/7 | 3.45E-02 | 1.76E-01 | ENO1 |
| Hyperosmotic Response (GO:0006972) | 1/7 | 3.45E-02 | 1.76E-01 | YBX3 |
| Positive Regulation Of Ryanodine-Sensitive Calcium-Release Channel Activity (GO:0060316) | 1/7 | 3.45E-02 | 1.76E-01 | CALM2 |
| Leukocyte Aggregation (GO:0070486) | 1/7 | 3.45E-02 | 1.76E-01 | CD44 |
| Postsynapse Organization (GO:0099173) | 1/7 | 3.45E-02 | 1.76E-01 | WNT5A |
| Myoblast Migration (GO:0051451) | 1/7 | 3.45E-02 | 1.76E-01 | ANXA1 |
| Regulation Of Apoptotic Cell Clearance (GO:2000425) | 1/7 | 3.45E-02 | 1.76E-01 | TGM2 |
| Regulation Of Attachment Of Mitotic Spindle Microtubules To Kinetochore (GO:1902423) | 1/7 | 3.45E-02 | 1.76E-01 | BIRC5 |
| Positive Regulation Of Transferase Activity (GO:0051347) | 3/143 | 3.50E-02 | 1.76E-01 | HSP90AA1;PCNA;HNRNPA2B1 |
| Glucose Metabolic Process (GO:0006006) | 2/59 | 3.52E-02 | 1.76E-01 | KRT17;ENO1 |
| Microtubule Cytoskeleton Organization Involved In Mitosis (GO:1902850) | 2/59 | 3.52E-02 | 1.76E-01 | STMN1;BIRC5 |
| protein-DNA Complex Assembly (GO:0065004) | 2/59 | 3.52E-02 | 1.76E-01 | NASP;HMGB2 |
| Actin Filament Organization (GO:0007015) | 3/144 | 3.56E-02 | 1.77E-01 | TPM3;TPM1;ALDOA |
| Positive Regulation Of Protein Kinase Activity (GO:0045860) | 3/145 | 3.63E-02 | 1.79E-01 | TPX2;HSP90AA1;CALM2 |
| Regulation Of Extrinsic Apoptotic Signaling Pathway (GO:2001236) | 2/60 | 3.63E-02 | 1.79E-01 | LGALS3;ITGA6 |
| Skeletal System Development (GO:0001501) | 3/149 | 3.88E-02 | 1.79E-01 | WNT5A;CD44;TGM2 |
| Negative Regulation Of DNA-binding Transcription Factor Activity (GO:0043433) | 3/149 | 3.88E-02 | 1.79E-01 | TMSB4X;TAX1BP1;CYP1B1 |
| Nucleic Acid Transport (GO:0050657) | 1/8 | 3.93E-02 | 1.79E-01 | HNRNPA2B1 |
| Positive Regulation Of ATP Biosynthetic Process (GO:2001171) | 1/8 | 3.93E-02 | 1.79E-01 | TMSB4X |
| Regulation Of Vesicle Fusion (GO:0031338) | 1/8 | 3.93E-02 | 1.79E-01 | ANXA1 |
| Collagen-Activated Signaling Pathway (GO:0038065) | 1/8 | 3.93E-02 | 1.79E-01 | ITGA2 |
| Sequestering Of Actin Monomers (GO:0042989) | 1/8 | 3.93E-02 | 1.79E-01 | TMSB4X |
| Skin Morphogenesis (GO:0043589) | 1/8 | 3.93E-02 | 1.79E-01 | ITGA6 |
| Positive Regulation Of Ligase Activity (GO:0051351) | 1/8 | 3.93E-02 | 1.79E-01 | TMSB4X |
| Positive Regulation Of Macrophage Cytokine Production (GO:0060907) | 1/8 | 3.93E-02 | 1.79E-01 | WNT5A |
| Tetrahydrofolate Interconversion (GO:0035999) | 1/8 | 3.93E-02 | 1.79E-01 | TYMS |
| Positive Regulation Of Megakaryocyte Differentiation (GO:0045654) | 1/8 | 3.93E-02 | 1.79E-01 | HMGB2 |
| Positive Regulation Of Mitochondrial Calcium Ion Concentration (GO:0051561) | 1/8 | 3.93E-02 | 1.79E-01 | TGM2 |
| Unsaturated Fatty Acid Biosynthetic Process (GO:0006636) | 1/8 | 3.93E-02 | 1.79E-01 | SCD |
| Vitamin K Metabolic Process (GO:0042373) | 1/8 | 3.93E-02 | 1.79E-01 | NQO1 |
| Positive Regulation Of Protein Kinase C Signaling (GO:0090037) | 1/8 | 3.93E-02 | 1.79E-01 | WNT5A |
| Protein Localization To Perinuclear Region Of Cytoplasm (GO:1905719) | 1/8 | 3.93E-02 | 1.79E-01 | SQSTM1 |
| Mesodermal Cell Differentiation (GO:0048333) | 1/8 | 3.93E-02 | 1.79E-01 | ITGA2 |
| Negative Regulation Of Chromosome Organization (GO:2001251) | 1/8 | 3.93E-02 | 1.79E-01 | TOP2A |
| Positive Regulation Of Mitotic Cell Cycle Phase Transition (GO:1901992) | 2/63 | 3.97E-02 | 1.80E-01 | ANXA1;CCND1 |
| Negative Regulation Of Cell Population Proliferation (GO:0008285) | 5/379 | 4.16E-02 | 1.80E-01 | FTH1;IGFBP3;WNT5A;HMGA1;CYP1B1 |
| Positive Regulation Of T Cell Proliferation (GO:0042102) | 2/65 | 4.20E-02 | 1.80E-01 | ANXA1;CD24 |
| Positive Regulation Of Cell Cycle (GO:0045787) | 2/65 | 4.20E-02 | 1.80E-01 | CCND1;CITED2 |
| Response To Reactive Oxygen Species (GO:0000302) | 2/66 | 4.32E-02 | 1.80E-01 | TPM1;SQSTM1 |
| Negative Regulation Of Necroptotic Process (GO:0060546) | 1/9 | 4.41E-02 | 1.80E-01 | YBX3 |
| Wnt Signaling Pathway Involved In Midbrain Dopaminergic Neuron Differentiation (GO:1904953) | 1/9 | 4.41E-02 | 1.80E-01 | WNT5A |
| Arachidonate Transport (GO:1903963) | 1/9 | 4.41E-02 | 1.80E-01 | ANXA1 |
| Arachidonic Acid Secretion (GO:0050482) | 1/9 | 4.41E-02 | 1.80E-01 | ANXA1 |
| Autophagy Of Peroxisome (GO:0030242) | 1/9 | 4.41E-02 | 1.80E-01 | SQSTM1 |
| Nucleobase-Containing Compound Biosynthetic Process (GO:0034654) | 1/9 | 4.41E-02 | 1.80E-01 | TYMS |
| Regulation Of Tau-Protein Kinase Activity (GO:1902947) | 1/9 | 4.41E-02 | 1.80E-01 | HSP90AA1 |
| Positive Regulation Of RNA Biosynthetic Process (GO:1902680) | 1/9 | 4.41E-02 | 1.80E-01 | TOP2A |
| Dopamine Transport (GO:0015872) | 1/9 | 4.41E-02 | 1.80E-01 | TGM2 |
| Ectoderm Development (GO:0007398) | 1/9 | 4.41E-02 | 1.80E-01 | ITGA6 |
| Skeletal Muscle Tissue Regeneration (GO:0043403) | 1/9 | 4.41E-02 | 1.80E-01 | ANXA1 |
| Positive Regulation Of Establishment Of Protein Localization To Telomere (GO:1904851) | 1/9 | 4.41E-02 | 1.80E-01 | CCT6A |
| Excitatory Synapse Assembly (GO:1904861) | 1/9 | 4.41E-02 | 1.80E-01 | WNT5A |
| Granulocyte Migration (GO:0097530) | 1/9 | 4.41E-02 | 1.80E-01 | ANXA1 |
| Homologous Recombination (GO:0035825) | 1/9 | 4.41E-02 | 1.80E-01 | NUCKS1 |
| Intestinal Cholesterol Absorption (GO:0030299) | 1/9 | 4.41E-02 | 1.80E-01 | LIMA1 |
| Positive Regulation Of Transcription Of Nucleolar Large rRNA By RNA Polymerase I (GO:1901838) | 1/9 | 4.41E-02 | 1.80E-01 | NCL |
| Postsynapse Assembly (GO:0099068) | 1/9 | 4.41E-02 | 1.80E-01 | WNT5A |
| Lipid Import Into Cell (GO:0140354) | 1/9 | 4.41E-02 | 1.80E-01 | ACSL3 |
| Long-Chain Fatty Acid Import Into Cell (GO:0044539) | 1/9 | 4.41E-02 | 1.80E-01 | ACSL3 |
| Quinone Metabolic Process (GO:1901661) | 1/9 | 4.41E-02 | 1.80E-01 | NQO1 |
| Regulation Of RNA Polymerase II Regulatory Region Sequence-Specific DNA Binding (GO:1903025) | 1/9 | 4.41E-02 | 1.80E-01 | TMSB4X |
| Regulation Of Animal Organ Morphogenesis (GO:2000027) | 1/9 | 4.41E-02 | 1.80E-01 | CITED2 |
| Negative Regulation Of Cell Adhesion Mediated By Integrin (GO:0033629) | 1/9 | 4.41E-02 | 1.80E-01 | CYP1B1 |
| Activation Of Cysteine-Type Endopeptidase Activity Involved In Apoptotic Process (GO:0006919) | 2/67 | 4.44E-02 | 1.80E-01 | CYCS;ANP32B |
| Regulation Of Neurogenesis (GO:0050767) | 2/67 | 4.44E-02 | 1.80E-01 | HMGB2;TGM2 |
| Positive Regulation Of Multicellular Organismal Process (GO:0051240) | 5/387 | 4.48E-02 | 1.81E-01 | SCD;CFL1;WNT5A;LCN2;ENO1 |
| Regulation Of Heart Contraction (GO:0008016) | 2/69 | 4.68E-02 | 1.81E-01 | TPM1;CALM2 |
| Phagocytosis (GO:0006909) | 2/69 | 4.68E-02 | 1.81E-01 | ANXA1;TGM2 |
| Response To Molecule Of Bacterial Origin (GO:0002237) | 2/69 | 4.68E-02 | 1.81E-01 | HMGB2;CD24 |
| Negative Regulation Of Extrinsic Apoptotic Signaling Pathway (GO:2001237) | 2/70 | 4.80E-02 | 1.81E-01 | LGALS3;ITGA6 |
| Neutrophil Chemotaxis (GO:0030593) | 2/70 | 4.80E-02 | 1.81E-01 | LGALS3;PPIA |
| Positive Regulation Of Protein Polymerization (GO:0032273) | 2/70 | 4.80E-02 | 1.81E-01 | HSP90AA1;PFN1 |
| Positive Regulation Of Protein Transport (GO:0051222) | 2/70 | 4.80E-02 | 1.81E-01 | ACSL3;PPIA |
| Regulation Of Dendritic Cell Differentiation (GO:2001198) | 1/10 | 4.89E-02 | 1.81E-01 | LGALS3 |
| Regulation Of Establishment Of Protein Localization To Telomere (GO:0070203) | 1/10 | 4.89E-02 | 1.81E-01 | CCT6A |
| Negative Regulation Of Pathway-Restricted SMAD Protein Phosphorylation (GO:0060394) | 1/10 | 4.89E-02 | 1.81E-01 | PMEPA1 |
| Negative Regulation Of Programmed Necrotic Cell Death (GO:0062099) | 1/10 | 4.89E-02 | 1.81E-01 | YBX3 |
| Regulation Of Lamellipodium Morphogenesis (GO:2000392) | 1/10 | 4.89E-02 | 1.81E-01 | CD44 |
| Regulation Of Mitotic Sister Chromatid Separation (GO:0010965) | 1/10 | 4.89E-02 | 1.81E-01 | BIRC5 |
| Regulation Of Peroxisome Proliferator Activated Receptor Signaling Pathway (GO:0035358) | 1/10 | 4.89E-02 | 1.81E-01 | CITED2 |
| Omega-Hydroxylase P450 Pathway (GO:0097267) | 1/10 | 4.89E-02 | 1.81E-01 | CYP1B1 |
| Regulation Of Protein Localization To Cajal Body (GO:1904869) | 1/10 | 4.89E-02 | 1.81E-01 | CCT6A |
| Removal Of Superoxide Radicals (GO:0019430) | 1/10 | 4.89E-02 | 1.81E-01 | NQO1 |
| Positive Regulation Of Attachment Of Spindle Microtubules To Kinetochore (GO:0051987) | 1/10 | 4.89E-02 | 1.81E-01 | BIRC5 |
| Signal Release (GO:0023061) | 1/10 | 4.89E-02 | 1.81E-01 | TGM2 |
| Urogenital System Development (GO:0001655) | 1/10 | 4.89E-02 | 1.81E-01 | ANXA1 |
| Hematopoietic Stem Cell Proliferation (GO:0071425) | 1/10 | 4.89E-02 | 1.81E-01 | WNT5A |
| Positive Regulation Of Protein Localization To Cajal Body (GO:1904871) | 1/10 | 4.89E-02 | 1.81E-01 | CCT6A |
| Midbrain Dopaminergic Neuron Differentiation (GO:1904948) | 1/10 | 4.89E-02 | 1.81E-01 | WNT5A |
| Mitotic Spindle Elongation (GO:0000022) | 1/10 | 4.89E-02 | 1.81E-01 | BIRC5 |
| Mitotic Spindle Midzone Assembly (GO:0051256) | 1/10 | 4.89E-02 | 1.81E-01 | BIRC5 |
| Muscle Filament Sliding (GO:0030049) | 1/10 | 4.89E-02 | 1.81E-01 | TPM1 |

**Supplemental Table 3. Feature Scores/Ranks per Method for Efficient Differentiation Dataset.** LASSO and RFR utilize the absolute value of the coefficient for ranking, and these values are shown below, aiming for 50 features. QUBO utilizes the negative cost function values, with corresponding ranks provided.

|  | LASSO | | RFR | | QUBO | |
| --- | --- | --- | --- | --- | --- | --- |
| Rank | Gene | Abs_Coef | Gene | Abs_Coef | Gene | QUBO_rank |
| 1 | THY1 | 0.2163 | DYNLT1 | 1.8961 | DYNLT1 | -0.1064 |
| 2 | KRT10 | 0.1669 | YWHAB | 1.6881 | YWHAB | -0.1001 |
| 3 | DYNLT1 | 0.1616 | THY1 | 1.6583 | ID1 | -0.0987 |
| 4 | ID1 | 0.1415 | CLDN7 | 1.3975 | APLN | -0.0927 |
| 5 | APLN | 0.1383 | ID1 | 1.3034 | THY1 | -0.0902 |
| 6 | GNG5 | 0.1365 | APLN | 1.1805 | CLDN7 | -0.0883 |
| 7 | TMA7 | 0.1252 | KRT10 | 1.0444 | EIF2S2 | -0.0749 |
| 8 | PDAP1 | 0.1246 | EVA1B | 0.9344 | EVA1B | -0.0714 |
| 9 | CLDN7 | 0.1144 | TRH | 0.8867 | TMA7 | -0.0669 |
| 10 | EIF2S2 | 0.1111 | MAP1B | 0.7678 | KRT10 | -0.0643 |
| 11 | ANP32E | 0.1107 | IGFBP5 | 0.6505 | TRH | -0.0578 |
| 12 | PRR13 | 0.1002 | PDAP1 | 0.6357 | PDAP1 | -0.0549 |
| 13 | KTN1 | 0.0973 | KLK10 | 0.6247 | FABP5 | -0.0532 |
| 14 | CDKN1C | 0.0960 | RPS19BP1 | 0.5904 | VIM | -0.0526 |
| 15 | HDAC3 | 0.0899 | EIF2S2 | 0.5730 | ID3 | -0.0515 |
| 16 | FABP5 | 0.0876 | KTN1 | 0.5532 | ATF5 | -0.0496 |
| 17 | ARL6IP4 | 0.0826 | TMA7 | 0.5238 | GNG5 | -0.0488 |
| 18 | BEX1 | 0.0824 | S100A3 | 0.5213 | RPS19BP1 | -0.0458 |
| 19 | SEC62 | 0.0803 | GDF15 | 0.5002 | ANP32E | -0.0456 |
| 20 | EIF4E2 | 0.0756 | ATF5 | 0.4842 | KLK10 | -0.0456 |
| 21 | LITAF | 0.0751 | VIM | 0.4766 | ARPC3 | -0.0442 |
| 22 | SFRP1 | 0.0743 | PCAT14 | 0.4548 | MAP1B | -0.0442 |
| 23 | ETV3L | 0.0738 | BEX1 | 0.4500 | POMP | -0.0441 |
| 24 | ARPC3 | 0.0734 | SH3BP5 | 0.4038 | ACTR2 | -0.0431 |
| 25 | SHC1 | 0.0673 | CDKN1C | 0.4034 | TUBA1C | -0.0407 |
| 26 | WDR83OS | 0.0655 | RHOC | 0.4014 | IGFBP5 | -0.0406 |
| 27 | RPS19BP1 | 0.0652 | ARPC3 | 0.3968 | S100A3 | -0.0399 |
| 28 | EVA1B | 0.0647 | TP53I11 | 0.3943 | HMGB3 | -0.0395 |
| 29 | GDF15 | 0.0642 | ADGRL4 | 0.3877 | SLC4A11 | -0.0385 |
| 30 | MGST3 | 0.0632 | TUBA1C | 0.3844 | PCAT14 | -0.0376 |
| 31 | BCL2L1 | 0.0594 | SLC4A11 | 0.3834 | PRR13 | -0.0369 |
| 32 | FLNC | 0.0583 | ISG20 | 0.3681 | LITAF | -0.0342 |
| 33 | STK4 | 0.0574 | HMGB3 | 0.3615 | MYL12B | -0.0338 |
| 34 | DUSP6 | 0.0562 | POMP | 0.3601 | IER2 | -0.0333 |
| 35 | HMGB3 | 0.0561 | FABP5 | 0.3496 | DRAP1 | -0.0319 |
| 36 | TMEM167A | 0.0552 | SFRP1 | 0.3429 | BEX1 | -0.0317 |
| 37 | ZFP36L2 | 0.0541 | PRTG | 0.3320 | XRCC5 | -0.0311 |
| 38 | PIK3R1 | 0.0530 | HDAC7 | 0.3286 | DPYSL2 | -0.0291 |
| 39 | ATP6V0B | 0.0527 | SOX17 | 0.3274 | SFRP1 | -0.0288 |
| 40 | PAPOLA | 0.0489 | SPCS3 | 0.3273 | SPCS3 | -0.0287 |
| 41 | TPM3 | 0.0483 | PLAUR | 0.3195 | EIF4EBP1 | -0.0284 |
| 42 | PDE4B | 0.0479 | CD81 | 0.3180 | TP53I11 | -0.0284 |
| 43 | CAST | 0.0475 | DUSP6 | 0.3141 | NTS | -0.0277 |
| 44 | DPYSL2 | 0.0473 | SYT1 | 0.3118 | NAP1L1 | -0.0276 |
| 45 | DPEP1 | 0.0473 | NTS | 0.3116 | GDF15 | -0.0276 |
| 46 | E2F4 | 0.0472 | SKIL | 0.3114 | RAMP2 | -0.0272 |
| 47 | TMBIM4 | 0.0471 | PDCD6 | 0.3088 | RGS10 | -0.0268 |
| 48 | SAV1 | 0.0470 | CLIC1 | 0.3065 | HMGN1 | -0.0264 |
| 49 | SOX17 | 0.0469 | ANP32E | 0.3044 | CD81 | -0.0244 |
| 50 | COX5B | 0.0463 | FGFR1 | 0.2992 | OAZ1 | -0.0232 |

**Supplemental Table 4. Feature Scores/Ranks per Method for Drug Resistance Dataset.** LASSO and RFR utilize the absolute value of the coefficient for ranking, and these values are shown below, aiming for 100 features. QUBO utilizes the negative cost function values, with corresponding ranks provided.

|  | LASSO | | RFR | | QUBO | |
| --- | --- | --- | --- | --- | --- | --- |
| Rank | Gene | Abs_Coef | Gene | Abs_Coef | Gene | QUBO_rank |
| 1 | SLC7A5 | 0.0269 | HMGA1 | 1.7553 | TUBA1B | -0.2452 |
| 2 | HMGA1 | 0.0250 | TUBA1B | 1.2858 | HMGA1 | -0.2212 |
| 3 | CYP1B1 | 0.0160 | SLC7A5 | 1.0215 | S100A6 | -0.1793 |
| 4 | S100A6 | 0.0151 | S100A6 | 0.9650 | EMP1 | -0.1665 |
| 5 | TPM1 | 0.0138 | EMP1 | 0.8281 | TACSTD2 | -0.1563 |
| 6 | TGM2 | 0.0130 | TPM1 | 0.7491 | SMC4 | -0.1474 |
| 7 | FADS2 | 0.0128 | TACSTD2 | 0.6229 | FTH1 | -0.1467 |
| 8 | ALDOA | 0.0120 | TOP2A | 0.6032 | NUCKS1 | -0.1426 |
| 9 | SLC3A2 | 0.0114 | IGFBP3 | 0.5854 | ITGA6 | -0.1418 |
| 10 | DBI | 0.0113 | TGM2 | 0.5604 | ENO1 | -0.1364 |
| 11 | PPIA | 0.0108 | NUCKS1 | 0.5509 | IGFBP3 | -0.1338 |
| 12 | FGFR1 | 0.0101 | MALAT1 | 0.5064 | MALAT1 | -0.1306 |
| 13 | EMP1 | 0.0092 | HMGB2 | 0.4997 | TPM1 | -0.1291 |
| 14 | CBX3 | 0.0079 | ITGA6 | 0.4975 | SLC7A5 | -0.1232 |
| 15 | PABPC3 | 0.0079 | CCDC80 | 0.4893 | HMGB2 | -0.1160 |
| 16 | TUBB6 | 0.0068 | KRT17 | 0.4563 | LGALS3 | -0.1152 |
| 17 | C4BPB | 0.0065 | SMC4 | 0.4561 | HSP90AA1 | -0.1133 |
| 18 | SCD | 0.0061 | FTH1 | 0.4508 | DTYMK | -0.1126 |
| 19 | CCDC80 | 0.0057 | SERPINE1 | 0.4470 | TGM2 | -0.1125 |
| 20 | MCAM | 0.0057 | CYP1B1 | 0.4018 | HNRNPA2B1 | -0.1078 |
| 21 | AURKB | 0.0053 | ANXA1 | 0.3980 | ANXA1 | -0.1067 |
| 22 | PTMS | 0.0046 | KRT19 | 0.3867 | PDLIM1 | -0.1044 |
| 23 | FASN | 0.0046 | FXYD3 | 0.3526 | CD24 | -0.1031 |
| 24 | FTL | 0.0045 | UBE2C | 0.3488 | TYMS | -0.1009 |
| 25 | PTMA | 0.0044 | CD24 | 0.3344 | FTL | -0.0994 |
| 26 | UBE2C | 0.0039 | PTTG1 | 0.3288 | CBX3 | -0.0970 |
| 27 | SERPINH1 | 0.0038 | PSMA7 | 0.3238 | GSTK1 | -0.0953 |
| 28 | CCNF | 0.0037 | WNT5A | 0.3229 | DBI | -0.0928 |
| 29 | NEGR1 | 0.0037 | PDLIM1 | 0.3196 | CCDC80 | -0.0922 |
| 30 | NQO1 | 0.0033 | CFL1 | 0.3167 | PTGR1 | -0.0906 |
| 31 | NPC2 | 0.0031 | YBX3 | 0.3034 | SLC3A2 | -0.0900 |
| 32 | NDC80 | 0.0030 | LCN2 | 0.3030 | DEK | -0.0896 |
| 33 | INSIG1 | 0.0030 | FSTL1 | 0.2978 | KRT19 | -0.0886 |
| 34 | NCAPG | 0.0029 | FTL | 0.2933 | ANLN | -0.0883 |
| 35 | MAL | 0.0029 | DTYMK | 0.2856 | TUBA1C | -0.0877 |
| 36 | SRRM2 | 0.0028 | AGR2 | 0.2735 | NPC2 | -0.0870 |
| 37 | CALM2 | 0.0028 | TPX2 | 0.2704 | SCD | -0.0859 |
| 38 | PSAT1 | 0.0027 | TYMS | 0.2693 | TPX2 | -0.0835 |
| 39 | SULF2 | 0.0026 | FASN | 0.2654 | PCNA | -0.0829 |
| 40 | ACSL3 | 0.0025 | SLC3A2 | 0.2628 | TOP2A | -0.0827 |
| 41 | RDH11 | 0.0025 | OLR1 | 0.2597 | ALDH1A3 | -0.0819 |
| 42 | SPARC | 0.0023 | FADS2 | 0.2575 | CALB2 | -0.0811 |
| 43 | FSTL1 | 0.0022 | PRC1 | 0.2565 | NOP56 | -0.0803 |
| 44 | FYN | 0.0021 | NPC2 | 0.2501 | ALDOA | -0.0803 |
| 45 | BEST1 | 0.0020 | CENPF | 0.2471 | TUBB4B | -0.0800 |
| 46 | FUCA2 | 0.0015 | DBI | 0.2455 | FSTL1 | -0.0788 |
| 47 | EPDR1 | 0.0014 | DEK | 0.2428 | ELF3 | -0.0784 |
| 48 | HSP90AA1 | 0.0014 | PTGR1 | 0.2424 | NCL | -0.0781 |
| 49 | SLC12A2 | 0.0013 | CBX3 | 0.2414 | PTMA | -0.0759 |
| 50 | SMS | 0.0013 | TUBA1C | 0.2403 | CD44 | -0.0755 |
| 51 | UBC | 0.0012 | ROMO1 | 0.2399 | ACSL3 | -0.0754 |
| 52 | S100A4 | 0.0012 | MT2A | 0.2373 | SQSTM1 | -0.0754 |
| 53 | BUD31 | 0.0012 | WFDC2 | 0.2366 | PSMA7 | -0.0753 |
| 54 | CDCA8 | 0.0011 | SCD | 0.2365 | PFN1 | -0.0743 |
| 55 | GNG12 | 0.0011 | ACKR3 | 0.2343 | LCN2 | -0.0741 |
| 56 | RAD21 | 0.0011 | ALDH1A3 | 0.2316 | BIRC5 | -0.0733 |
| 57 | HMGA2 | 0.0011 | HNRNPA2B1 | 0.2278 | WNT5A | -0.0731 |
| 58 | SLIRP | 0.0010 | PLK1 | 0.2268 | TMSB4X | -0.0722 |
| 59 | HMGB2 | 0.0009 | ACSL3 | 0.2261 | KRT17 | -0.0722 |
| 60 | CNBP | 0.0009 | MIF | 0.2236 | CFL1 | -0.0721 |
| 61 | FBLIM1 | 0.0008 | KRT13 | 0.2224 | KRT13 | -0.0719 |
| 62 | MT1E | 0.0008 | GUK1 | 0.2217 | CITED2 | -0.0718 |
| 63 | MCM2 | 0.0007 | GULP1 | 0.2175 | GPRC5A | -0.0717 |
| 64 | SPNS1 | 0.0007 | SQSTM1 | 0.2172 | ALDH3A1 | -0.0704 |
| 65 | CD46 | 0.0006 | PCNA | 0.2155 | S100A4 | -0.0704 |
| 66 | ANO1 | 0.0006 | ENO1 | 0.2146 | PSAP | -0.0688 |
| 67 | FDPS | 0.0005 | NASP | 0.2117 | S100A2 | -0.0685 |
| 68 | TNFRSF12A | 0.0005 | LGALS3 | 0.2094 | MUC20 | -0.0681 |
| 69 | B3GNT3 | 0.0004 | ITGA2 | 0.2055 | NEAT1 | -0.0680 |
| 70 | TOP2A | 0.0004 | FYN | 0.2046 | HMGN2 | -0.0675 |
| 71 | CD44 | 0.0004 | CALB2 | 0.2037 | FXYD3 | -0.0671 |
| 72 | INCENP | 0.0003 | PHGDH | 0.2029 | CYCS | -0.0661 |
| 73 | XPOT | 0.0003 | TFPI2 | 0.2028 | ALPP | -0.0658 |
| 74 | S100A2 | 0.0002 | KLK7 | 0.2021 | ITGA2 | -0.0653 |
| 75 | C3 | 0.0002 | S100A2 | 0.2010 | STMN1 | -0.0645 |
| 76 | WWC1 | 0.0002 | GTSE1 | 0.2005 | TPM3 | -0.0645 |
| 77 | PRDX5 | 0.0002 | SKAP2 | 0.2004 | FADS2 | -0.0631 |
| 78 | ARHGEF26 | 0.0001 | ANP32B | 0.1999 | CYP1B1 | -0.0627 |
| 79 | GCSH | 0.0001 | NME1 | 0.1992 | TFPI2 | -0.0622 |
| 80 | PCDH1 | 0.0001 | YPEL3 | 0.1973 | USP1 | -0.0615 |
| 81 | MSANTD3 | 4.46E-05 | SNRPB | 0.1969 | SLIRP | -0.0614 |
| 82 | RPS6KL1 | 3.41E-05 | HMMR | 0.1935 | CCND1 | -0.0607 |
| 83 | MAP1B | 2.94E-05 | GOLGB1 | 0.1929 | PNRC1 | -0.0599 |
| 84 | FGF2 | 2.60E-05 | CDKN3 | 0.1927 | NASP | -0.0549 |
| 85 | IGFN1 | 2.39E-05 | QSOX1 | 0.1926 | PMEPA1 | -0.0533 |
| 86 | LRRFIP1 | 1.73E-05 | CAPN8 | 0.1917 | WFDC2 | -0.0518 |
| 87 | SLC39A10 | 3.98E-06 | PSAT1 | 0.1898 | BEST1 | -0.0517 |
| 88 |  |  | PFN1 | 0.1894 | LIMA1 | -0.0513 |
| 89 |  |  | POLR2F | 0.1887 | CALM2 | -0.0511 |
| 90 |  |  | ST13 | 0.1883 | EEF1A1 | -0.0507 |
| 91 |  |  | ZWINT | 0.1880 | TAX1BP1 | -0.0506 |
| 92 |  |  | SLC25A5 | 0.1878 | RHOBTB3 | -0.0504 |
| 93 |  |  | USP8 | 0.1863 | YBX3 | -0.0491 |
| 94 |  |  | PSAP | 0.1862 | ANP32B | -0.0488 |
| 95 |  |  | CYCS | 0.1859 | CAPN8 | -0.0483 |
| 96 |  |  | SEC62 | 0.1853 | CCT6A | -0.0471 |
| 97 |  |  | SLC2A4RG | 0.1845 | PPIA | -0.0344 |
| 98 |  |  | EEF2 | 0.1839 | NQO1 | -0.0340 |
| 99 |  |  | SF3B1 | 0.1832 | EIF5B | -0.0319 |
| 100 |  |  | NEK2 | 0.1830 | CSTB | -0.0308 |
